# Efficacy and Safety of Normal Saline Injection for Corticosteroid‐Induced Cutaneous Atrophy: A Systematic Review

**DOI:** 10.1111/jocd.70986

**Published:** 2026-06-09

**Authors:** Jia Qi Adam Bai, Jennifer V. H. Tran, Chaocheng Liu

**Affiliations:** ^1^ Faculty of Medicine University of Ottawa Ottawa Ontario Canada; ^2^ Schulich School of Medicine and Dentistry Western University London Ontario Canada; ^3^ Department of Dermatology and Skin Science University of British Columbia Vancouver British Columbia Canada; ^4^ School of Medicine Simon Fraser University Surrey British Columbia Canada

**Keywords:** corticosteroid‐induced cutaneous atrophy, intralesional corticosteroids, lipoatrophy, normal saline injection, systematic review

To the Editor,

Corticosteroid‐induced cutaneous atrophy is a common iatrogenic adverse event characterized by dermal thinning, lipoatrophy, and visible contour deformities that can be cosmetically distressing and slow to resolve [1, 2]. Normal saline (NS) injections have been increasingly reported as a simple, accessible, and minimally invasive treatment option, although their efficacy and safety remain insufficiently established. This systematic review summarizes the evidence on the efficacy and safety of NS injections in the management of corticosteroid‐induced cutaneous atrophy.

This systematic review was conducted in accordance with PRISMA guidelines and was prospectively registered on PROSPERO (CRD420251274107). MEDLINE, Embase, Web of Science, and CENTRAL were searched from inception to January 5, 2026, using predefined keywords (Table S1). Quality of evidence was assessed using the Oxford Centre for Evidence‐Based Medicine 2011 Levels of Evidence. Studies were independently screened by two reviewers. A total of 16 articles reporting on 41 patients were included (Figure 1; Table S2). Risk of bias was assessed using the Joanna Briggs Institute tool; 11 (68.8%) studies were low risk while 5 (31.3%) were moderate (Table S3).

**FIGURE 1:**
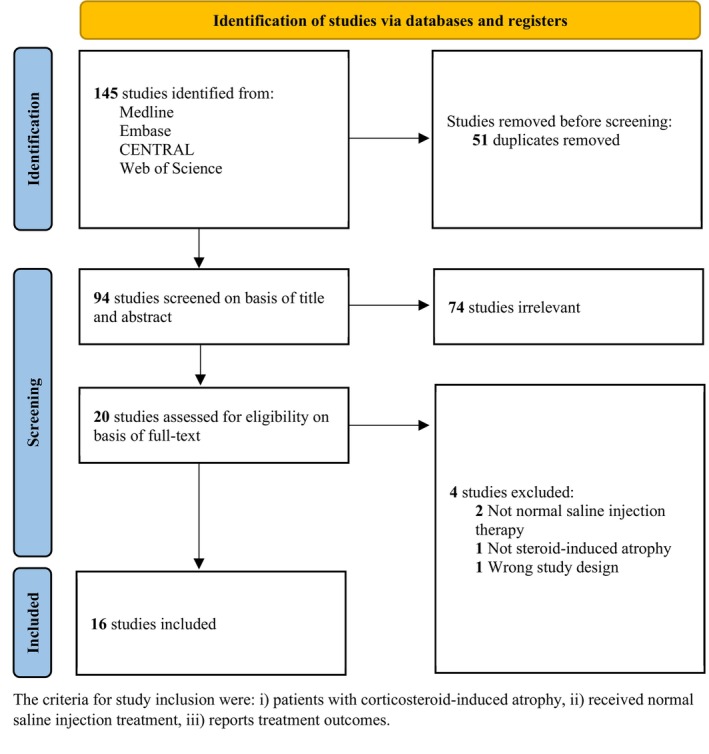
Flow diagram of literature screening using the Preferred Reporting Items for Systematic Reviews and Meta‐Analyses (PRISMA) guidelines. Figure adapted from http://prisma‐statement.org.

Among the 41 patients, the mean age was 20.2 ± 15.8 years, 58.5% (24/41) were females, and a total of 48 lesions (mean: 1.2 ± 0.5) were reported. The most common indications for corticosteroid treatment were airway obstructive disease (25.0%, 6/28), eczema (16.7%, 4/24), and cysts (16.7%, 4/24) (Table S2). Reported corticosteroids administered were predominantly triamcinolone acetonide (TA; 85.3%, 29/34; intralesional [*n* = 20], intramuscular [*n* = 7], periarticular [*n* = 1], peri‐tendinous [*n* = 1]), followed by dexamethasone (11.8%, 4/34; intramuscular [*n* = 4]), and hydrocortisone (2.9%, 1/34; intramuscular [*n* = 1]). The mean time from steroid exposure to atrophy was 10.3 ± 12.7 weeks. Atrophy presentations included lipoatrophy (61.0%, 24/41), dermal atrophy (12.2%, 5/41), and mixed atrophy (26.8%, 11/41). Mean lesion size was 1.8 cm × 2.5 cm (range: 0.3 × 0.4 to 4.0 × 7.0), with the most common sites being the buttocks (50.0%, 24/48) and deltoid (8.3%, 4/48). Of the 41 patients, 23 (56.1%) had concomitant hypopigmentation associated with corticosteroid‐injection.

NS 0.9% was administered intralesionally in all patients (Tables 1 and S2). Mean NS injection volume per session was 12.9 ± 18.7 mL and mean frequency was 4.2 ± 1.9 sessions. Mean treatment duration was 8.2 ± 5.4 weeks, with dosing intervals ranging from once weekly to once every 4 weeks. Of the 48 lesions, 83.3% (40/48) achieved complete resolution, 8.3% (4/48) achieved partial resolution, and 8.3% (4/48) achieved no response. Of 11 patients with hypopigmentation outcome data, 90.9% (10/11) reported improvement in pigmentation. Adverse events were reported in one (2.4%) patient, which included injection‐site discomfort and ecchymosis. No recurrence of corticosteroid‐induced cutaneous atrophy was reported after a mean follow‐up duration of 3.7 ± 3.4 months.

**TABLE 1:** Reported treatment parameters for intralesional normal saline injection in corticosteroid‐induced cutaneous atrophy.

Reported parameters	Intralesional normal saline injection
Sample size (*n*, lesions)	41 patients, 48 lesions
Normal saline concentration	0.9%
Mean injected volume per session, mL (mean ± SD)	12.9 ± 18.7
Injected volume per session, mL (range)	1.0–100.0
Mean number of treatment sessions (mean ± SD)	4.2 ± 1.9
Number of treatment sessions (range)	1.0–9.0
Treatment interval, weeks (range)	1.0–4.0
Mean treatment duration, weeks (mean ± SD)	8.2 ± 5.4
Treatment duration, weeks (range)	1.0–36.0

Based on the available literature, intralesional 0.9% NS injections administered at a mean volume of 12.9 mL per session over 4.2 treatment sessions spaced 1–4 weeks apart resulted in complete resolution in 83.3% of lesions. The pathogenesis of corticosteroid‐induced cutaneous atrophy involves local inhibition of fibroblast proliferation as well as reduced collagen and glycosaminoglycan synthesis, resulting in dermal and subcutaneous volume loss [2]. NS injections may address this process by mechanically resuspending persistent corticosteroid crystals within atrophic tissue [1, 3]. This redistribution facilitates the clearance of residual steroid deposits and reduces local corticosteroid activity, thereby allowing restoration of normal dermal architecture and adipose volume [1, 4]. Our findings suggest that intralesional NS injections represent a safe, low‐cost, and minimally invasive therapeutic option for patients with corticosteroid‐induced cutaneous atrophy. Study limitations include heterogeneity in treatment protocols and outcome reporting, potential reporting bias, and the possibility of spontaneous resolution, limiting attribution of treatment response to NS injections alone. Future prospective studies are warranted to establish standardized treatment protocols and to better characterize long‐term outcomes.

## Funding

The authors have nothing to report.

## Ethics Statement

An ethics statement is not applicable because this study is based exclusively on published literature.

## Consent

The authors have nothing to report.

## Conflicts of Interest

The authors declare no conflicts of interest.

## Supporting information


**Table S1:** Search strategy used for literature screening.
**Table S2:** Clinical characteristics and treatment outcomes of patients with corticosteroid‐induced cutaneous atrophy treated with intralesional normal saline injections.
**Table S3:** Joanna Briggs Institute (JBI) risk of bias assessment for included studies (*n* = 16).

## Data Availability

The data underlying this article are available in the article and in its online Supporting Information.

## References

[1] P. R. Shumaker , J. Rao , and M. P. Goldman , “Treatment of Local, Persistent Cutaneous Atrophy Following Corticosteroid Injection With Normal Saline Infiltration,” Dermatologic Surgery 31, no. 10 (2005): 1340–1343, 10.1111/j.1524-4725.2005.31216. 16188192

[2] E. Niculet , C. Bobeica , and A. L. Tatu , “Glucocorticoid‐Induced Skin Atrophy: The Old and the New,” Clinical, Cosmetic and Investigational Dermatology 13 (2020): 1041–1050, 10.2147/CCID.S224211. 33408495 PMC7779293

[3] M. A. Shiffman , “New Treatment of Steroid‐Induced Fat Atrophy,” Plastic and Reconstructive Surgery 109, no. 7 (2002): 2609–2610, 10.1097/00006534-200206000-00090. 12045618

[4] A. Oikarinen and P. Autio , “New Aspects of the Mechanism of Corticosteroid‐Induced Dermal Atrophy,” Clinical and Experimental Dermatology 16, no. 6 (1991): 416–419, 10.1111/j.1365-2230.1991.tb01225.x. 1806315

