# Supplementary material for: Efficacy and Safety of Normal Saline Injection for Corticosteroid‐Induced Cutaneous Atrophy: A Systematic Review
Source: J Cosmet Dermatol. 2026 Jun 9;25(6):e70986. doi: 10.1111/jocd.70986 (PMC13250237; doi:10.1111/jocd.70986)
Supplement: Supplementary file 1 — Table S1: Search strategy used for literature screening. Table S2: Clinical characteristics and treatment outcomes of patients with corticosteroid‐induced cutaneous atrophy treated with intralesional normal saline injections. Table S3: Joanna Briggs Institute (JBI) risk of bias assessment for included studies (n = 16). [file JOCD-25-e70986-s001.docx]

**Supplemental Table 1.** Search strategy used for literature screening.

Ovid MEDLINE(R) ALL <1946 to January 5, 2026>

| **#** | **Search line** | **Results** |
| --- | --- | --- |
| 1 | "steroid*".ab,kf,ti. | 295303 |
| 2 | "corticosteroid*".ab,kf,ti. | 140004 |
| 3 | triamcinolone.ab,kf,ti. | 10189 |
| 4 | "intralesional corticosteroid*".ab,kf,ti. | 721 |
| 5 | "topical corticosteroid*".ab,kf,ti. | 7817 |
| 6 | kenalog.ab,kf,ti. | 253 |
| 7 | atroph*.ab,kf,ti. | 158138 |
| 8 | lipoatroph*.ab,kf,ti. | 1890 |
| 9 | cutaneous atroph*.ab,kf,ti. | 248 |
| 10 | skin atroph*.ab,kf,ti. | 852 |
| 11 | subcutaneous atroph*.ab,kf,ti. | 87 |
| 12 | fat atroph*.ab,kf,ti. | 269 |
| 13 | "hypopigment*".ab,kf,ti. | 5085 |
| 14 | "depressed contour*".ab,kf,ti. | 5 |
| 15 | normal saline.ab,kf,ti. | 28459 |
| 16 | "saline injection*".ab,kf,ti. | 5540 |
| 17 | intralesional saline.ab,kf,ti. | 26 |
| 18 | sodium chloride.ab,kf,ti. | 23191 |
| 19 | "NS injection*".ab,kf,ti. | 64 |
| 20 | infiltration saline.ab,kf,ti. | 1 |
| 21 | 1 or 2 or 3 or 4 or 5 or 6 | 419068 |
| 22 | 7 or 8 or 9 or 10 or 11 or 12 or 13 or 14 | 164446 |
| 23 | 15 or 16 or 17 or 18 or 19 or 20 | 56469 |
| 24 | 21 and 22 and 23 | 28 |

Embase Classic+Embase <1947 to 2026 January 5>

| **#** | **Search line** | **Results** |
| --- | --- | --- |
| 1 | "steroid*".ab,kf,ti. | 478320 |
| 2 | "corticosteroid*".ab,kf,ti. | 238497 |
| 3 | triamcinolone.ab,kf,ti. | 15039 |
| 4 | "intralesional corticosteroid*".ab,kf,ti. | 1131 |
| 5 | "topical corticosteroid*".ab,kf,ti. | 13076 |
| 6 | kenalog.ab,kf,ti. | 536 |
| 7 | atroph*.ab,kf,ti. | 255303 |
| 8 | lipoatroph*.ab,kf,ti. | 2762 |
| 9 | cutaneous atroph*.ab,kf,ti. | 403 |
| 10 | skin atroph*.ab,kf,ti. | 1432 |
| 11 | subcutaneous atroph*.ab,kf,ti. | 144 |
| 12 | fat atroph*.ab,kf,ti. | 406 |
| 13 | "hypopigment*".ab,kf,ti. | 7926 |
| 14 | "depressed contour*".ab,kf,ti. | 8 |
| 15 | normal saline.ab,kf,ti. | 51364 |
| 16 | "saline injection*".ab,kf,ti. | 8407 |
| 17 | intralesional saline.ab,kf,ti. | 37 |
| 18 | sodium chloride.ab,kf,ti. | 31021 |
| 19 | "NS injection*".ab,kf,ti. | 113 |
| 20 | 1 or 2 or 3 or 4 or 5 or 6 | 682434 |
| 21 | 7 or 8 or 9 or 10 or 11 or 12 or 13 or 14 | 264824 |
| 22 | 15 or 16 or 17 or 18 or 19 | 89383 |
| 23 | 20 and 21 and 22 | 67 |

Cochrane CENTRAL

| **#** | **Search line** | **Results** |
| --- | --- | --- |
| 1 | "steroid*".ab,kf,ti. | 30196 |
| 2 | "corticosteroid*".ab,kf,ti. | 26553 |
| 3 | triamcinolone.ab,kf,ti. | 3731 |
| 4 | "intralesional corticosteroid*".ab,kf,ti. | 106 |
| 5 | "topical corticosteroid*".ab,kf,ti. | 2175 |
| 6 | kenalog.ab,kf,ti. | 101 |
| 7 | atroph*.ab,kf,ti. | 8916 |
| 8 | lipoatroph*.ab,kf,ti. | 217 |
| 9 | cutaneous atroph*.ab,kf,ti. | 28 |
| 10 | skin atroph*.ab,kf,ti. | 211 |
| 11 | subcutaneous atroph*.ab,kf,ti. | 5 |
| 12 | fat atroph*.ab,kf,ti. | 26 |
| 13 | "hypopigment*".ab,kf,ti. | 352 |
| 14 | normal saline.ab,kf,ti. | 19973 |
| 15 | "saline injection*".ab,kf,ti. | 1281 |
| 16 | intralesional saline.ab,kf,ti. | 20 |
| 17 | sodium chloride.ab,kf,ti. | 3413 |
| 18 | "NS injection*".ab,kf,ti. | 27 |
| 19 | infiltration saline.ab,kf,ti. | 1 |
| 20 | 1 or 2 or 3 or 4 or 5 or 6 | 54045 |
| 21 | 7 or 8 or 9 or 10 or 11 or 12 or 13 | 9408 |
| 22 | 14 or 15 or 16 or 17 or 18 or 19 | 24071 |
| 23 | 20 and 21 and 22 | 15 |

Web of Science

| **#** | **Search line** | **Results** |
| --- | --- | --- |
| 1 | TS=((steroid* OR corticosteroid* OR triamcinolone OR "intralesional corticosteroid*" OR "topical corticosteroid*" OR kenalog) AND (atroph* OR lipoatroph* OR "cutaneous atroph*" OR "skin atroph*" OR "subcutaneous atroph*" OR "fat atroph*" OR hypopigment* OR "depressed contour*") AND ("normal saline" OR "saline injection*" OR "intralesional saline" OR "sodium chloride" OR "NS injection*" OR "infiltration saline")) | 35 |

**Supplemental Table 2.** Clinical characteristics and treatment outcomes of patients with corticosteroid-induced cutaneous atrophy treated with intralesional normal saline injections. AE, adverse event; CAR, case report; CAS, case series; CR, complete resolution; F, female; IL, intralesional; IM, intramuscular; M, male; NOR, no response; NR, not reported; NS, normal saline; PR, partial resolution; QW, once weekly; Q2W, once every 2 weeks; Q3W, once every 3 weeks; Q4W, once every 4 weeks; RCS, retrospective cohort study; RCT, randomized controlled trial.

| **Study** | **Study design (evidence level)** | **Sample size (n)** | **Mean patient age (years)** | **Sex (M/F)** | **Corticosteroid indication (n)** | **Corticosteroid treatment [route, dose, frequency] (n)** | **Number of lesions per patient (n)** | **Lesion site (n)** | **Lesion size (n)** | **Time from steroid exposure to atrophy (weeks)** | **Atrophy type (n)** | **NS injection Treatment [route, NS concentration, volume injected, frequency, N sessions] (n)** | **Outcome**  **(CR, PR, NOR) (n)** | **Therapy duration (weeks)** | **Follow-up duration (months)** | **AEs (n)** |
| --- | --- | --- | --- | --- | --- | --- | --- | --- | --- | --- | --- | --- | --- | --- | --- | --- |
| Birnbaum, 2020^1^ | CAR (4) | 1 | 53 | F (1) | Medial epicondylitis (1) | Triamcinolone acetonide [peri-tendinous, 40 mg/mL, once] (1) | 1 | Left medial epicondyle (1) | NR | 14 | Lipoatrophy (1) | Normal saline [IL, 0.9%, 5 mL, every 10 days, 6] (1) | CR (1) | 8.0 | 4.5 | NR |
| Biswas, 2023^2^ | CAS (4) | 1 of 9 | 2.9 | M (1) | Airway obstructive disease (1) | Dexamethasone [IM, NR, NR] (1) | 1 | Left buttock (1) | 1 cm x 1 cm (1) | 3.5 | Mixed (1) | Normal saline [IL, 0.9%, 10-15 mL, Q2W, 4] (1) | CR (1) | 8.0 | 2.0 | NR |
| Biswas, 2023^2^ | CAS (4) | 1 of 9 | 10 | M (1) | Eczema (1) | Triamcinolone acetonide [IM, NR, NR] (1) | 1 | Right deltoid (1) | 1.5 cm x 1 cm (1) | 6 | Mixed (1) | Normal saline [IL, 0.9%, 10-15 mL, Q2W, 4] (1) | NOR (1) | 8.0 | 2.0 | NR |
| Biswas, 2023^2^ | CAS (4) | 1 of 9 | 2 | F (1) | Airway obstructive disease (1) | Dexamethasone [IM, NR, NR] (1) | 1 | Right buttock (1) | 2 cm x 1.5 cm (1) | 3 | Mixed (1) | Normal saline [IL, 0.9%, 10-15 mL, Q2W, 4] (1) | CR (1) | 8.0 | 2.0 | NR |
| Biswas, 2023^2^ | CAS (4) | 1 of 9 | 2.5 | M (1) | Eczema (1) | Triamcinolone acetonide [IM, NR, NR] (1) | 1 | Left deltoid (1) | 2 cm x 2 cm (1) | 4 | Mixed (1) | Normal saline [IL, 0.9%, 10-15 mL, Q2W, 4] (1) | NOR (1) | 8.0 | 2.0 | NR |
| Biswas, 2023^2^ | CAS (4) | 1 of 9 | 1.9 | M (1) | Eczema (1) | Triamcinolone acetonide [IM, NR, NR] (1) | 1 | Left deltoid (1) | 1 cm x 1.5 cm (1) | 2 | Mixed (1) | Normal saline [IL, 0.9%, 10-15 mL, Q2W, 4] (1) | CR (1) | 8.0 | 2.0 | NR |
| Biswas, 2023^2^ | CAS (4) | 1 of 9 | 5 | F (1) | Airway obstructive disease (1) | Dexamethasone [IM, NR, NR] (1) | 1 | Right triceps (1) | 1.5 cm x 1 cm (1) | 6 | Mixed (1) | Normal saline [IL, 0.9%, 10-15 mL, Q2W, 4] (1) | NOR (1) | 8.0 | 2.0 | NR |
| Biswas, 2023^2^ | CAS (4) | 1 of 9 | 3 | M (1) | Eczema (1) | Triamcinolone acetonide [IM, NR, NR] (1) | 1 | Left deltoid (1) | 1 cm x 1 cm (1) | 3 | Mixed (1) | Normal saline [IL, 0.9%, 10-15 mL, Q2W, 4] (1) | CR (1) | 8.0 | 2.0 | NR |
| Biswas, 2023^2^ | CAS (4) | 1 of 9 | 5 | F (1) | NR (1) | Triamcinolone acetonide [IM, NR, NR] (1) | 1 | Left arm (1) | 1.5 cm x 2 cm (1) | 4 | Mixed (1) | Normal saline [IL, 0.9%, 10-15 mL, Q2W, 4] (1) | CR (1) | 8.0 | 2.0 | NR |
| Biswas, 2023^2^ | CAS (4) | 1 of 9 | 3.5 | F (1) | Airway obstructive disease (1) | Triamcinolone acetonide [IM, NR, NR] (1) | 1 | Left buttock (1) | 2 cm x 2 cm (1) | 2 | Mixed (1) | Normal saline [IL, 0.9%, 10-15 mL, Q2W, 4] (1) | CR (1) | 8.0 | 2.0 | NR |
| Crowe, 2022^3^ | CAR (4) | 1 | 20 | F (1) | Migraines (1) | Steroid [IL, NR, NR] (1) | 3 | Forehead (2), occipital scalp (1) | NR | 8 | Mixed (1) | Normal saline [IL, 0.9%, NR, QW, 6] (1) | PR (1) | 6.0 | 1.5 | NR |
| Daruwalla, 2020^4^ | CAR (4) | 1 | 7 | M (1) | Fever with rash (1) | Triamcinolone acetonide [IL, NR, NR] (1) | 1 | Right buttock (1) | NR | 4 | Lipoatrophy (1) | Normal saline [IL, 0.9%, 10-12 mL, Q2W, 4] (1) | CR (1) | 8.0 | 4.0 | NR |
| DeJulio, 2021^5^ | CAR (4) | 1 | 35 | M (1) | Chronic left groin pain (1) | Triamcinolone acetonide [IL, 40 mg/mL, once] (1) | 1 | Left inguinal region | 2 cm x 3 cm (1) | 6 | Lipoatrophy (1) | Normal saline [IL, 0.9%, 3-10 mL, Q2W, 9] (1) | CR (1) | 18.0 | 5.5 | NR |
| Dhinsa, 2021^6^ | CAR (4) | 1 | 24 | F (1) | Keloid from umbilicus piercing (1) | Triamcinolone acetonide [IL, 40 mg/mL, Q2M] (1) | 1 | Umbilicus (1) | NR | 8 | Mixed (1) | Normal saline [IL, 0.9%, 3-10 mL, Q2W-Q4W, 6] (1) | CR (1) | 18.0 | 10.5 | Discomfort (1), ecchymosis (1) |
| Lin, 2023^7^ | CAR (4) | 1 | 50 | F (1) | Hip bursitis pain (1) | Triamcinolone acetonide [periarticular, 80 mg, once] (1) | 1 | Right greater trochanter (1) | 4 cm x 6 cm (1) | 52 | Lipoatrophy (1) | Normal saline [IL, 0.9%, NR, Q2W, 6] (1) | NOR (1) | 1.0 | 12.0 | NR |
| Lo, 2008^8^ | CAS (4) | 1 of 2 | 8 | F (1) | Urticaria (1) | Triamcinolone acetonide [IL, NR, Q2W] (1) | 1 | Right buttock (1) | 6 cm x 4 cm (1) | 8 | Lipoatrophy (1) | Normal saline [IL, 0.9%, NR, Q2W, 6] (1) | PR (1) | 12.0 | 12.0 | NR |
| Lo, 2008^8^ | CAS (4) | 2 of 2 | NR | M (1) | Urticaria (1) | Triamcinolone acetonide [IL, NR, Q2W] (1) | 1 | Right buttock (1) | 7 cm x 4 cm (1) | 8 | Lipoatrophy (1) | Normal saline [IL, 0.9%, NR, Q2W, 6] (1) | PR (1) | 12.0 | 12.0 | NR |
| Margulies, 2015^9^ | CAR (4) | 1 | 51 | F (1) | Fibromyalgia (1) | Steroid [IL, NR, NR] (1) | 3 | Left scapula (1), right ankle (1), right sacrum (1) | NR | 52 | Lipoatrophy (1) | Normal saline [IL, 0.9%, 3-12 mL Q4W, 9] (1) | CR (1) | 36.0 | 9.0 | NR |
| Sadati, 2018^10^ | CAS (4) | 1 of 2 | 4 | F (1) | Allergic reactions (1), asthma (1) | Hydrocortisone [IM, NR, NR] (1) | 1 | Right buttock (1) | NR | 8 | Lipoatrophy (1) | Normal saline [IL, 0.9%, 7-17 mL, Q2W, 6] (1) | CR (1) | 12.0 | 6.0 | NR |
| Sadati, 2018^10^ | CAS (4) | 2 of 2 | 14 | F (1) | Acne vulgaris (1) | Triamcinolone acetonide [IL, NR, NR] (1) | 1 | Right buttock (1) | NR | 4 | Lipoatrophy (1) | Normal saline [IL, 0.9%, 8-15 mL, Q2W, 6] (1) | CR (1) | 12.0 | 6.0 | NR |
| Sharma, 2022^11^ | RCS (3) | 1 of 12 | 20 | M (1) | NR (1) | Triamcinolone acetonide [IL, NR, NR] (1) | 1 | Right buttock (1) | 4 cm x 3 cm (1) | 7 | Lipoatrophy (1) | Normal saline [IL, 0.9%, NR, Q2W, 2] (1) | CR (1) | 4.0 | 1.0 | NR |
| Sharma, 2022^11^ | RCS (3) | 2 of 12 | 32 | F (1) | NR (1) | Triamcinolone acetonide [IL, NR, NR] (1) | 1 | Left buttock (1) | 2.5 cm x 2 cm (1) | 4.5 | Lipoatrophy (1) | Normal saline [IL, 0.9%, NR, Q2W, 3] (1) | CR (1) | 6.0 | 1.5 | NR |
| Sharma, 2022^11^ | RCS (3) | 3 of 12 | 42 | F (1) | NR (1) | Triamcinolone acetonide [IL, NR, NR] (1) | 1 | Right buttock (1) | 4 cm x 3.5 cm (1) | 6 | Lipoatrophy (1) | Normal saline [IL, 0.9%, NR, Q2W, 3] (1) | CR (1) | 6.0 | 1.5 | NR |
| Sharma, 2022^11^ | RCS (3) | 4 of 12 | 25 | M (1) | NR (1) | Triamcinolone acetonide [IL, NR, NR] (1) | 1 | Left buttock (1) | 3.5 cm x 2 cm (1) | 7 | Lipoatrophy (1) | Normal saline [IL, 0.9%, NR, Q2W, 4] (1) | CR (1) | 8.0 | 2.0 | NR |
| Sharma, 2022^11^ | RCS (3) | 5 of 12 | 22 | F (1) | NR (1) | Triamcinolone acetonide [IL, NR, NR] (1) | 1 | Right buttock (1) | 1.5 cm x 1.5 cm (1) | 7 | Lipoatrophy (1) | Normal saline [IL, 0.9%, NR, Q2W, 3] (1) | CR (1) | 6.0 | 1.5 | NR |
| Sharma, 2022^11^ | RCS (3) | 6 of 12 | 4 | M (1) | NR (1) | Triamcinolone acetonide [IL, NR, NR] (1) | 1 | Right buttock (1) | 5 cm x 3.5 cm (1) | 5 | Lipoatrophy (1) | Normal saline [IL, 0.9%, NR, Q2W, 2] (1) | CR (1) | 4.0 | 1.0 | NR |
| Sharma, 2022^11^ | RCS (3) | 7 of 12 | 26 | M (1) | NR (1) | Triamcinolone acetonide [IL, NR, NR] (1) | 1 | Left buttock (1) | 3.5 cm x 3 cm (1) | 10 | Lipoatrophy (1) | Normal saline [IL, 0.9%, NR, Q2W, 4] (1) | CR (1) | 8.0 | 2.0 | NR |
| Sharma, 2022^11^ | RCS (3) | 8 of 12 | 25 | F (1) | NR (1) | Triamcinolone acetonide [IL, NR, NR] (1) | 1 | Left buttock (1) | 2 cm x 1.5 cm (1) | 9 | Lipoatrophy (1) | Normal saline [IL, 0.9%, NR, Q2W, 2] (1) | CR (1) | 4.0 | 1.0 | NR |
| Sharma, 2022^11^ | RCS (3) | 9 of 12 | 45 | F (1) | NR (1) | Triamcinolone acetonide [IL, NR, NR] (1) | 1 | Right buttock (1) | 2 cm x 2 cm (1) | 8 | Lipoatrophy (1) | Normal saline [IL, 0.9%, NR, Q2W-Q4W, 5] (1) | CR (1) | 12.0 | 3.0 | NR |
| Sharma, 2022^11^ | RCS (3) | 10 of 12 | 21 | M (1) | NR (1) | Triamcinolone acetonide [IL, NR, NR] (1) | 1 | Right buttock (1) | 1.5 cm x 3 cm (1) | 8 | Lipoatrophy (1) | Normal saline [IL, 0.9%, NR, Q2W, 4] (1) | CR (1) | 8.0 | 2.0 | NR |
| Sharma, 2022^11^ | RCS (3) | 11 of 12 | 35 | F (1) | NR (1) | Triamcinolone acetonide [IL, NR, NR] (1) | 1 | Left buttock (1) | 2.5 cm x 1.5 cm (1) | 5 | Lipoatrophy (1) | Normal saline [IL, 0.9%, NR, Q2W, 2] (1) | CR (1) | 4.0 | 1.0 | NR |
| Sharma, 2022^11^ | RCS (3) | 12 of 12 | 22 | M (1) | NR (1) | Triamcinolone acetonide [IL, NR, NR] (1) | 1 | Right buttock (1) | 2 cm x 2 cm (1) | 6 | Lipoatrophy (1) | Normal saline [IL, 0.9%, NR, Q2W, 4] (1) | CR (1) | 8.0 | 2.0 | NR |
| Shiffman, 2002^12^ | CAR (4) | 1 | NR | F (1) | Prevent recurrence of hypertrophic scars after breast saline implants (1) | Steroid [IL, NR, NR] (1) | 2 | Left breast (1), right breast (1) | NR | NR | Lipoatrophy (1) | Normal saline [IL, 0.9%, 100 mL, once, 1) (1) | CR (1) | 4.0 | 1.0 | NR |
| Shiver, 2017^13^ | CAR (4) | 1 | NR | F (1) | Epidermoid cyst (1) | Triamcinolone acetonide [IL, 1 mg/mL, once] (1) | 1 | Left cheek (1) | NR | 3 | Lipoatrophy (1) | Normal saline [IL, 0.9%, NR, once, 1) (1) | CR (1) | 8.0 | 2.0 | NR |
| Shumaker, 2005^14^ | CAS (4) | 1 of 4 | 44 | F (1) | Cyst (1) | Steroid [IL, NR, NR] (1) | 1 | Chin (1) | 0.9 cm x 0.25 cm (1) | 3 | Dermal atrophy (1) | Normal saline [IL, 0.9%, 5 mL, QW, 3) (1) | CR (1) | 3.0 | 1.0 | NR |
| Shumaker, 2005^14^ | CAS (4) | 2 of 4 | 18 | M (1) | Acneiform cyst (1) | Steroid [IL, NR, NR] (1) | 1 | Right medial forehead (1) | 0.8 cm x 0.25 cm (1) | 32 | Dermal atrophy (1) | Normal saline [IL, 0.9%, 5 mL, QW, 4) (1) | CR (1) | 4.0 | 1.25 | NR |
| Shumaker, 2005^14^ | CAS (4) | 3 of 4 | 31 | F (1) | Cyst (1) | Steroid [IL, NR, NR] (1) | 1 | Right cheek (1) | 1.8 cm x 0.25 cm (1) | 2 | Dermal atrophy (1) | Normal saline [IL, 0.9%, 5 mL, QW, 5) (1) | CR (1) | 5.0 | 1.25 | NR |
| Shumaker, 2005^14^ | CAS (4) | 4 of 4 | 27 | F (1) | Asthma (1) | Steroid [IL, NR, NR] (1) | 1 | Left lateral hip (1) | 0.4 cm x 0.3 cm (1) | 1 | Dermal atrophy (1) | Normal saline [IL, 0.9%, 10 mL, QW, 6) (1) | CR (1) | 6.0 | 4.5 | NR |
| Tiwary, 2020^15^ | CAS (4) | 1 of 2 | 5 | F (1) | Respiratory problems (1) | Dexamethasone [IM, NR, NR] (1) | 2 | Left buttock (1), right buttock (1) | 1.5 cm x 1 cm (2) | 20 | Lipoatrophy (1) | Normal saline [IL, 0.9%, 1 mL, Q2W, 3) (1) | CR (1) | 6.0 | 6.0 | NR |
| Tiwary, 2020^15^ | CAS (4) | 2 of 2 | 4 | M (1) | Allergic reactions (1) | Triamcinolone acetonide [IM, NR, NR] (1) | 2 | Left buttock (1), right buttock (1) | 1.5 cm x 1 cm (2) | 16 | Lipoatrophy (1) | Normal saline [IL, 0.9%, 1 mL, Q2W, 3) (1) | CR (1) | 6.0 | 6.0 | NR |
| Vos, 2023^16^ | CAR (4) | 1 | 16 | M (1) | Hypertrophic scar (1) | Triamcinolone acetonide [IL, 40 mg/mL, once] (1) | 1 | Left superomedial knee (1) | NR | 48 | Dermal atrophy (1) | Normal saline [IL, 0.9%, 10 mL, once, 1) (1) | CR (1) | 1.0 | 8.0 | NR |

**References**

1. Birnbaum A, Yoon MY, Struhl S. Serial saline solution injections for the treatment of lipoatrophy and depigmentation after corticosteroid injection for medial epicondylitis. *JSES Int*. 2020;4(4):1002-1005. doi:10.1016/j.jseint.2020.08.009

2. Biswas R, Sarkar D, Ghoshal L. Normal saline infiltration as a treatment of steroid injection induced tissue atrophy: A clinical study. *Biomedicine*. 2023;43(5):1472-1475. doi:10.51248/.v43i5.2719

3. Crowe S, Schmidgal EC, Dermatologist, Naval Medical Center San Diego, San Diego, California. Intradermal Injection of Normal Saline for Treatment of Fat Atrophy Following Corticosteroid Injection. *Consultant*. Published online 2022. doi:10.25270/con.2022.02.00008

4. Daruwalla SB, Dhurat RS, Sharma A, Nitya MSN, Ghate S. Reversal of steroid‐induced lipoatrophy with serial injections of isotonic saline in a child. *Dermatol Ther*. 2020;33(3). doi:10.1111/dth.13354

5. DeJulio PA, Vallabh JC, Sopkovich JA. Treatment of Steroid-Induced Lipoatrophy Following Transverse Abdominis Plane Block With Serial Intralesional Isotonic Saline Injections: A Case Report. *AA Pract*. 2021;15(8):e01509. doi:10.1213/XAA.0000000000001509

6. Dhinsa H, McGuinness AE, Ferguson NN. Successful treatment of corticosteroid-induced cutaneous atrophy and dyspigmentation with intralesional saline in the setting of keloids. *JAAD Case Rep*. 2021;16:116-119. doi:10.1016/j.jdcr.2021.08.022

7. Lin LJ, Chen TX, Jazrawi LM, Chiu ES. Severe Contour Deformity of the Hip Following Corticosteroid Injection. *Bull Hosp Jt Dis 2013*. 2023;81(4):285-288.

8. Lo LK, Hung CM, Tsai TF. Successful treatment of two cases of localised involutional lipoatrophy with intralesional normal saline. *J Paediatr Child Health*. 2008;44(12):749-751. doi:10.1111/j.1440-1754.2008.01416.x

9. Margulies SL, Morris A. Successful treatment of lipoatrophy with normal saline. *JAAD Case Rep*. 2015;1(6):415-417. doi:10.1016/j.jdcr.2015.10.008

10. Sadati MS, Boroujeni NH, Sepaskhah M. Normal saline injection; a promising method to treat steroid- induced atrophy. *Iranian Journal of Dermatology*. 2018;21(1):20-22.

11. Sharma RK, Gupta M, Rani R. Delineating Injectable Triamcinolone-Induced Cutaneous Atrophy and Therapeutic Options in 24 Patients—A Retrospective Study. *Indian Dermatol Online J*. 2022;13(2):199-206. doi:10.4103/idoj.idoj_483_21

12. Shiffman MA. New treatment of steroid-induced fat atrophy. *Plast Reconstr Surg*. 2002;109(7):2609-2610. doi:10.1097/00006534-200206000-00090

13. Shiver M. Treatment of intralesional corticosteroid-induced lipoatrophy of the face with serial saline injections: My personal experience. *J Am Acad Dermatol*. 2017;76(6):AB266. doi:10.1016/j.jaad.2017.04.1033

14. Shumaker PR, Rao J, Goldman MP. Treatment of local, persistent cutaneous atrophy following corticosteroid injection with normal saline infiltration. *Dermatol Surg Off Publ Am Soc Dermatol Surg Al*. 2005;31(10):1340-1343. doi:10.1111/j.1524-4725.2005.31216

15. Tiwary PK, Gurudiwan P, Chaudhary R. Normal saline used in successful treatment of localised lipoatrophy in children. *Indian J Clin Exp Dermatol*.

16. Vos A, Ringin S, Chong AH, Robertson SJ. Delayed linear lipoatrophy following corticosteroid injection of a hypertrophic scar: Successfully reversed by isotonic saline injection. *Australas J Dermatol*. 2023;64(4). doi:10.1111/ajd.14135

**Supplemental Table 3.** Joanna Briggs Institute (JBI) risk of bias assessment for included studies (n = 16).

| **COHORT STUDIES (n = 1)** | | | | | | | | | | | | | |
| --- | --- | --- | --- | --- | --- | --- | --- | --- | --- | --- | --- | --- | --- |
| **Author, Year** | **Q1** | **Q2** | **Q3** | **Q4** | **Q5** | **Q6** | **Q7** | **Q8** | **Q9** | **Q10** | **Q11** | **% Yes** | **Overall risk*** |
| Sharma, 2022 | Y | Y | Y | Y | N | Y | N | Y | N | N | Y | 63.6 | Moderate |

| **CASE REPORTS (n = 10)** | | | | | | | | | | |
| --- | --- | --- | --- | --- | --- | --- | --- | --- | --- | --- |
| **Author, Year** | **Q1** | **Q2** | **Q3** | **Q4** | **Q5** | **Q6** | **Q7** | **Q8** | **% Yes** | **Overall risk** |
| Birnbaum, 2020 | Y | Y | Y | Y | Y | Y | Y | Y | 100.0 | Low |
| Crowe, 2022 | Y | Y | Y | Y | Y | Y | N | Y | 87.5 | Low |
| Daruwalla, 2020 | Y | Y | Y | Y | Y | Y | N | Y | 87.5 | Low |
| DeJulio, 2021 | Y | Y | Y | Y | Y | Y | Y | Y | 100.0 | Low |
| Dhinsa, 2021 | Y | Y | Y | Y | Y | Y | Y | Y | 100.0 | Low |
| Lin, 2023 | Y | Y | Y | Y | Y | Y | N | Y | 87.5 | Low |
| Margulies, 2015 | Y | Y | Y | Y | Y | Y | Y | Y | 100.0 | Low |
| Shiffman, 2002 | N | N | Y | N | Y | Y | N | Y | 50.0 | Moderate |
| Shiver, 2017 | N | Y | Y | N | Y | Y | N | Y | 62.5 | Moderate |
| Vos, 2023 | Y | Y | Y | Y | Y | Y | N | Y | 87.5 | Low |

| **CASE SERIES (n = 5)** | | | | | | | | | | | | |
| --- | --- | --- | --- | --- | --- | --- | --- | --- | --- | --- | --- | --- |
| **Author, Year** | **Q1** | **Q2** | **Q3** | **Q4** | **Q5** | **Q6** | **Q7** | **Q8** | **Q9** | **Q10** | **% Yes** | **Overall risk** |
| Biswas, 2023 | Y | N | Y | N | N | Y | Y | Y | Y | Y | 70.0 | Low |
| Lo, 2008 | Y | Y | Y | Y | N | N | Y | Y | Y | N | 70.0 | Low |
| Sadati, 2018 | Y | N | Y | N | N | Y | Y | Y | Y | N | 60.0 | Moderate |
| Shumaker, 2005 | Y | Y | Y | N | N | Y | Y | Y | Y | Y | 80.0 | Low |
| Tiwary, 2020 | N | Y | Y | N | N | Y | Y | Y | Y | N | 60.0 | Moderate |

Abbreviations: NA: Not applicable; N: no; Y: yes

*Studies were classified as low risk of bias if achieving a “yes” rating on 70% of applicable checklist items or above and moderate if between 50% and 70%.
